# A systematic review on clinical effectiveness, side-effect profile and meta-analysis on continuation rate of etonogestrel contraceptive implant

**DOI:** 10.1186/s12978-020-01054-y

**Published:** 2021-01-06

**Authors:** Kusum V. Moray, Himanshu Chaurasia, Oshima Sachin, Beena Joshi

**Affiliations:** 1 Regional Resource Hub for Health Technology Assessment, Indian Council of Medical Research, National Institute for Research in Reproductive Health, Jehangir Merwanji Street, Parel, Mumbai 400012 India; 2 Health Technology Assessment Secretariat, Department of Health Research, Ministry of Health and Family Welfare, New Delhi, India

**Keywords:** Etonogestrel subdermal contraceptive implant, Clinical effectiveness, Systematic review, Continuation rate

## Abstract

**Background:**

Unintended pregnancies (UIP) have a significant impact on health of women and the health budget of countries. Contraception is an effective way to prevent UIPs. The study objective was to collate evidence on clinical effectiveness of etonogestrel subdermal implant (ESI), continuation rate and side effect profile among eligible women of reproductive age group, as compared to levonorgestrel intrauterine system (LNG-IUS), copper intrauterine device (Cu-IUD) and depot medroxy progesterone acetate injections; other types of contraceptive implants were excluded as comparators.

**Methods:**

The protocol of the systematic review was registered in Prospero (registration number: CRD42018116580). MEDLINE via PubMed, Cochrane library and web of science were the electronic databases searched. A search strategy was formulated and studies from 1998 to 2019 were included. Clinical trial registries and grey literature search was done. Critical assessment of included studies was done using appropriate tools. A qualitative synthesis of included studies was done and a meta-analysis was conducted in RevMan software for continuation rates of ESI as compared to other long acting reversible contraceptives (LARC) e.g. LNG IUS and Cu-IUD.

**Results:**

The search yielded 23,545 studies. After excluding 467 duplicates, 23,078 titles were screened and 51 studies were included for the review. Eight of the 15 studies reporting clinical effectiveness reported 100% effectiveness and overall pearl index ranged from 0 to 1.4. One-year continuation rates ranged from 57–97%; 44–95% at the end of second year and 25–78% by 3 years of use. Abnormal menstruation was the most commonly reported side effect. There was no significant difference in bone mineral density at 1 year follow-up. The meta-analyses showed that odds ratio (OR) of 1-year continuation rate was 1.55 (1.36, 1.76) for LNG-IUS vs. ESI and 1.34 (1.13, 1.58) for copper-IUD vs. ESI; showing that continuation rates at the end of one-year were higher in LNG-IUS and copper-IUD as compared to ESI.

**Conclusion:**

ESI is clinically effective and safe contraceptive method to use, yet 1-year continuation rates are lower as compared to LNG-IUS and copper-IUD, mostly attributed to the disturbances in the menstruation.

## Plain English summary

The choice of becoming pregnant and timing it right in the life course of women depends on multiple biological, social and psychological factors. With improving access to contraception, in an ideal scenario, every pregnancy should be wanted and planned. However unplanned pregnancies still occur and some of the reasons are limited choice, inadequate access to contraceptive methods and their side effects. Unintended pregnancies have a large impact not only on the physical health of women (unsafe abortions and maternal deaths), but also on their social and psychological make-up. The burden of unintended pregnancies is very high at about 87 million a year. Improving the basket of choice of contraceptives is known to reduce unintended pregnancies. Etonogestrel implant is one such contraceptive that has not been available through government supported family planning programs in some countries including India. This implant contains a synthetic hormone called ‘etonogestrel’ packaged inside a special plastic rod, the size of a small matchstick. It can be placed under the skin of the arm (subdermal) and left in place for three years to prevent pregnancy by slow release of the hormone. Our research aimed at compiling evidence on this contraceptive implant using a systematic search strategy to assess how well it works, how many women continue using it for the prescribed period of three years and what were the side-effects. We found that ESI worked very well, it prevented pregnancies to a great extent, but due to its side-effects especially abnormal menstrual bleeding, a range of 30–70% women did not use the method for the complete 3-year period.

## Background

Unintended pregnancies are a major concern in our world, due to the psychological and economic distress they cause. About 45% of all pregnancies were unwanted/unintended in the years 2010–2014 [1]. They have a large impact on health of women due to unsafe abortions and maternal deaths [2]. Contraception, if used appropriately, prevents unintended pregnancies [3] and their consequences. One of the relatively newer methods of contraception is the contraceptive implant. Studies show that the etonogestrel implant is effective, yet there is evidence that the side-effect profile of the implant results in high rates of discontinuation. This systematic review aimed to collate evidence on clinical effectiveness, continuation rates and side-effect profile of the etonogestrel subdermal contraceptive implant (ESI), as a part of a health technology assessment (HTA). ESI is a long acting reversible contraceptive method that contains 68 mg of Etonogestrel. It is flexible and measures four centimetres in length and two millimetres in breadth; it is inserted under the skin of the upper arm of a woman, by a trained health care provider. Once inserted sub dermally in the arm, it can be left in place for three years; removal needs a small surgical incision. Etonogestrel is an artificial active metabolite of the synthetic progestin called ‘Desogestrel’ [4]. Its contraceptive effect is mainly because it prevents the release of luteinizing hormone (LH) and hence prevents ovulation. It thickens the cervical mucus and this reduces the entry of spermatozoa. It also modifies the endometrium and inhibits implantation of the fertilized ovum [5]. The previous generation of this implant was called “Implanon”. However the improvised version Nexplanon/Implanon-NXT additionally contains barium, rendering it radio-opaque, and a differently designed insertion device. An addendum to a clinical guideline for long acting reversible contraceptive (LARC) by United Kingdom National Institute for Health and Care excellence (UK NICE) had a consensus that as Nexplanon/Implanon-NXT was bioequivalent to Implanon, the literature on Implanon was considered to update the guidelines on LARC implants [6]. ESI is indicated in women who are not pregnant and are of reproductive age (15–45 years) who wish to prevent pregnancy. According to the World Health Organization (WHO) medical eligibility criteria, it is contraindicated in women with history of deep vein thrombosis, severe liver disease and breast cancer [7]. The objectives of this systematic review were to estimate incidence of unintended pregnancy/contraceptive failure rate, continuation rate and side-effects of ESI as compared with levonorgestrel intrauterine system, copper intrauterine device, and depot medroxy progesterone acetate.

## Methods

The protocol of this systematic review was registered and is available on Prospero (CRD42018116580, https://www.crd.york.ac.uk/prospero/display_record.php?ID=CRD42018116580). In this review we included studies that fit the inclusion criteria defined below and studies that were in English language. The population of interest for this review were women of reproductive age group 15–49 years who were eligible for contraception. The intervention of interest in this systematic review was etonogestrel sub dermal contraceptive implant (ESI). The comparators considered were copper Intra uterine devices (IUD), levonorgestrel IUS and contraceptive injectable (depot medroxy progesterone acetate i.e. DMPA). Studies with at least one of these as comparators or no comparator were included. The systematic review included studies published between January 1998 and December 2019 and grey literature. The study designs that were included were randomized control trials, non-randomized trials and observational studies. Observational studies included cross-sectional, case–control and cohort studies. The primary outcome that was assessed for effectiveness of the etonogestrel sub dermal contraceptive implant was unintended pregnancy rate/failure rate/pearl index while secondary outcomes included side effects/adverse events and continuation/discontinuation rates.

### Operational definitions used in this review

#### Long acting reversible contraceptive (LARC)

Contraceptive methods that require administration less than once per menstrual cycle or month [8].

#### Contraceptive failure

A contraceptive failure is defined as a conception that occurred during a month in which a woman (or her partner) was using a contraceptive method, as long as she did not report that she (or he) had stopped use before having become pregnant [9].

#### Contraceptive continuation rate

The cumulative probability that acceptors of a contraceptive method will still be using any contraceptive method after a specified period of time (e.g., 1 year) [10].

#### Contraceptive discontinuation

Starting contraceptive use and then stopping before the specified period for any reason while still at risk of an unintended pregnancy.

#### Pearl index

Number of pregnancies per 100 woman-years (WY) of contraceptive use [11].

#### Randomized control trial

The randomised control trial (RCT) is a research study design in which subjects are randomly assigned to one of two groups: one (the experimental group) receiving the intervention that is being tested, and the other (the comparison group or control) receiving an alternative (conventional) treatment. The two groups are then followed up to see if there are any differences between them in outcome [12].

#### Unintended pregnancy

Unintended pregnancies are pregnancies that are reported to have been either unwanted i.e., they occurred when no children, or no more children, were desired or mistimed i.e., they occurred earlier than desired [13].

### Exclusion criteria

We excluded studies that focussed on acceptability/feasibility /factors affecting use/utilization alone and abstracts in other languages. We excluded studies that had a different type of subdermal contraceptive implant as comparator, as this was not the purview of the health technology assessment under which this systematic review was conducted. Studies that focussed on specific timing of insertion of implant (like post-abortion or post-partum) and specific sub-groups like obese, human immunodeficiency virus (HIV) positive women etc. were excluded. The studies that looked at sub-group populations specifically were excluded because this review was meant to be applicable to the general population of reproductive age women.

### Search strategy

A list of Medical subject headings (MeSH) and free text search words was made by conducting a preliminary search of relevant studies. The electronic databases that were searched were MEDLINE via PubMed, Cochrane library and Web of Science. Four clinical trial registries (Clinical trial registry of National institutes of Health [14], Clinical trials registry of India [15], EU clinical trials registry [16], The Australian New Zealand Clinical Trials Registry (ANZCTR [17]) were searched for relevant clinical trials. Grey literature search included unpublished conference abstracts, unpublished dissertations/thesis, government reports, government documents and product-related documents on ProQuest and Open Grey online platforms. The search strategy was developed using appropriate Boolean terms and the MEDLINE via PubMed strategy is enclosed in Additional file 1. The search strategy was adapted as per the specific norms of each separate electronic database. The reference list of all selected studies for critical appraisal was hand-searched for other relevant studies for the systematic review. The search was conducted by two researchers independently. All abstracts were reviewed and those fitting into the inclusion criteria were selected after excluding duplicates. Any disagreement between the two researchers was settled by a third researcher with mutual consensus.

### Data collection and quality assessment strategy

The search was documented as per PRISMA guidelines [18]. A structured data extraction tool was used to extract the data. Methods, participant characteristics, interventions, comparators and outcomes were documented. Studies to be included for data extraction were assessed for quality. The quality of included studies in terms of methodology was critically assessed by two independent reviewers. Cochrane risk of bias tool [19] was used for randomized control trials; the risk of bias in non-randomized studies—of interventions (ROBINS-I) assessment tool was used to appraise non-randomized trials [20]; CASP checklist was used for cohort studies [21] and AXIS tool was used for cross-sectional studies [22]. The CASP checklist had 11 items on it. The tool does not have a scoring system; we hence used the following scoring to appraise the cohort studies: ≤ 2 negative attributes (no/can’t say) signified good quality; 3–5 negative attributes signified fair quality and ≥ 6 were marked as poor quality. The AXIS tool for cross sectional studies did not have a scoring system either, we used the following scoring method: of the 20 items on the tool; if ≤ 3 had negative attributes, it was marked as good quality; 4–8 was marked as fair quality and ≥ 9 was marked as poor quality.

### Data analysis

A qualitative synthesis of included studies was done and is presented as text and summarized using tables. The narrative results compare and evaluate the study designs used and the key results among included studies. Analysis of heterogeneity was done based on study designs, comparators and outcomes reported among the included studies. The studies reporting clinical effectiveness and adverse effects were not eligible for meta-analysis due to different or no comparators. Clinical effectiveness was reported as pearl index i.e. pregnancies per woman-years of use, where data was available. Side-effects were reported as percentages. Meta-analyses were conducted using RevMan software to compare the one-year continuation rates of ESI with LNG-IUS and Copper-IUD. The summary measure in the meta-analysis was odds ratio. Percentages of continuation rates in ESI and LNG-IUS were extracted from included studies, entered in RevMan software to derive the pooled odds ratio. The forest plot and I^2^, to denote dispersion of effect sizes of included studies, generated from the software were reported. Reporting bias for these comparisons was assessed by constructing funnel plots.

## Results

**Fig. 1:**
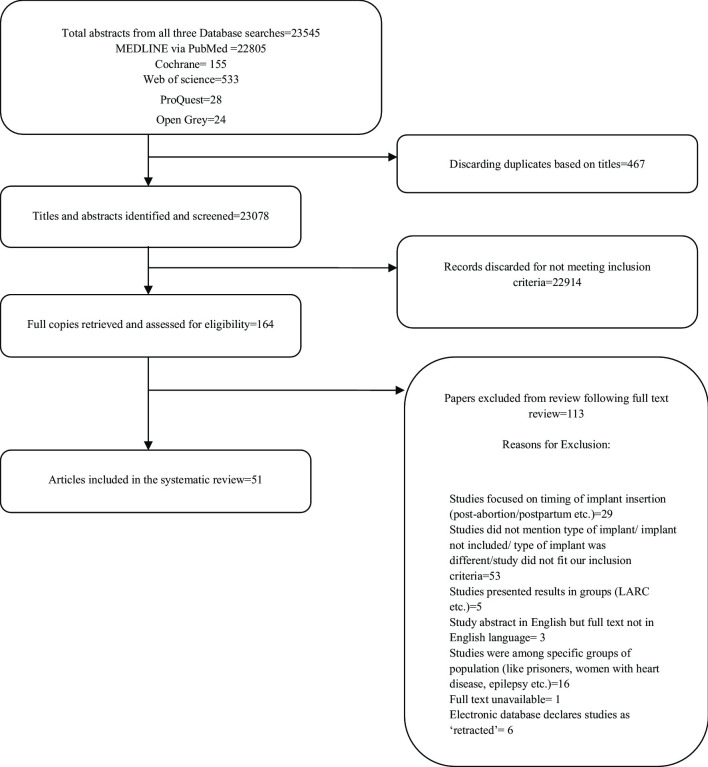
Flow chart of systematic review search and study selection. The flow chart is as per PRISMA guidelines for systematic reviews and shows the number of studies at each step of the review. Source: Constructed by authors

The summary of the search is presented as a PRISMA flow chart in Fig. 1. The search yielded a total of 23,545 titles from the three electronic databases and grey literature. Among these, 467 titles were duplicates and subsequently 23,078 abstracts were screened. 164 full text articles were retrieved as they seemed to fit the inclusion criteria. Of these, 113 were excluded. Three of the studies had abstracts in English language but had full text in a language other than English; hence they were excluded. 29 studies were excluded because they focussed on specific timing of insertion of implant, for instance among women immediately post-abortion and post-partum. Studies that did not mention type of implant/ studied a different type of implant were excluded. Studies that focussed on specific sub-groups of population like prisoners, obese/overweight women, women with heart disease or epilepsy etc. were excluded. Six studies were excluded as the electronic database stated them as ‘inappropriate data collection’ in a narrative review on implanon [23].

ProQuest and Open Grey forums were searched for unpublished articles. Key words from the search strategy were used in these websites. 28 working papers and conference titles were retrieved in ProQuest and 24 titles were available on Open Grey. None of the titles retrieved on ProQuest fit the inclusion criteria and were hence excluded. The titles on Open Grey were all in French and were hence excluded. A government of India report on an unpublished study on implanon was accessible to us. It was an executive summary of a Phase-III Multi-center clinical trial on ESI from India. 3119 women followed up for 93,617 women months showed that effectiveness was 100% and the overall continuation rate was 66.1 per 100 users at the end of 3 years. Frequent prolonged bleeding in women decreased over the three year duration of use from 11.8% at 3 months to 6.4% women at 36 months and amenorrhea increased from 11.6% at 3 months to 24.9% at 36 months [24].However all relevant data was not available to include the study in this review. Finally, 51 articles were included that addressed one or more of the outcomes; of which sixteen reported effectiveness of ESI. Side-effects of ESI were reported by 21 (41.2%) studies and 38 (74.5%) studies reported the continuation of implant by users in different parts of the world. The summary of findings of the 51 studies is presented in Table 1. Sixteen (31.4%) of the 51 studies reported more than one of the aforementioned outcomes. Most studies (28, 53.8%) compared ESI to other contraceptive methods. Five (9.8%) of the 51 studies were interventional trials, the rest were observational studies; five (9.8%) were cross-sectional and 30 (58.8%) were cohort studies (21 were prospective and 9 were retrospective). Eleven of the 51 (21.6%) studies were retrospective chart reviews. Of the 51 studies, five were drawn from the secondary analysis of the contraceptive CHOICE study. This was a prospective cohort study that promoted the use of long acting reversible methods in a region in the United States of America. Of the 51 studies, majority 34 (66.7%) were from high-income countries; 13 (25.5%) were from middle-income countries and 3(5.9%) in low income countries. One of the studies was a multi-country study.

**Table 1:** Table describing salient features of included studies

Author, country and year	Type of study	sample size	Comparator (s)	Effectiveness	Effect(s) reported frequency (percentage)	Continuation rate reported	Follow-up period	Reference number
Study design: cohort study								
Abraham M et al., USA, 2015	CHOICE cohort study	n = 6106	Cu-IUD, Age and parity	NA	NA	ENG implant continuation rate varied from 83 to 92% at 1 year and from 59 to 72% at 2 years. Nulliparous female were more likely to discontinue their LARC method among implant users adjusted HR 1.89, 95% CI 1.35–2.64	1, 2 years	[53]
Peipert et al., USA, 2011	CHOICE cohort study	n = 5087	LNG IUS, Cu-IUD and Etonogestrel implant	NA	NA	Continuation rate at one year for ENG implant was 83%	1 year	[63]
Neil O callahan et al., USA, 2013	CHOICE cohort study	n = 9256	LNG IUS, Cu-IUD and Etonogestrel implant	NA	NA	24-month continuation rate for implant was 69%	2 years	[72]
Grunloh et al., USA, 2013	CHOICE cohort study	n = 6167	LNG IUS, Cu-IUD and Etonogestrel implant	NA	NA	Discontinuation rates was 6.9% for the implant	6 months	[59]
Vickery et al., USA, 2013	CHOICE cohort study	n = 427	DMPA, LNG-IUS, Cu-IUD, ENG-implant	NA	No difference in weight gain	NA	1 year	[45]
Agostini et al., France, 2018	Retrospective cohort study	n = 42,365	COCs, progestin-only oral contraceptives, Cu-IUD, LNG-IUS and ENG implant	NA	NA	83.6% for the ENG implant	Up to 2 years	[54]
Berenson et al., USA, 2014	Retrospective study	LNG-IUS (n = 79,920) or ENG implants (n = 7374) inserted	LNG-IUS and ENG implant	98.821% for ESI and 98.8713% in LNG-IUS	The most frequent complications with both methods were related to abnormal menstruation, which was more likely to occur among ENG implant users	14.82% of ENG implant users discontinued within 1 year of insertion	1 year	[38]
Bitzer et al., Switzerland, 2004	Retrospective study	n = 1183	None	NA	Side-effects (visit 1) included infrequent bleeding (28%), amenorrhea (33%), prolonged bleeding (15%), and metromenorrhagia (frequent and heavy bleeding) (16%). Other reported side-effects at visit 1 included dizziness (12%), acne (11%), mood swings (8%) and headache (5%)	Implanon was removed prematurely in 235 women − 24%		[41]
Chiles et al., USA, 2016	Retrospective cohort study	> 1.7 million women, aged 14–40 years	LNG IUS, Cu-IUD and Etonogestrel implant	NA	NA	Among women who selected the implant, 32.0% continued their method at 36 months	3 years	[57]
Sara e casey, Democratic Republic of Congo, 2017	Retrospective cohort study	n = 548	Implant, IUD, Injection and Pill	NA	NA	Discontinuation rate at one year for LARC users(IUD and implant) was 13.9%	1 year	[42]
Thamkantho et al., Thailand, 2008	Retrospective clinic based study	n = 166	None	0 pregnancy in 1 year of use	30.3% had regular menstrual flow for a few months alternately with no menses for a few months; prolonged menstrual bleeding most common	NA	1 year	[33]
Teunissen et al., Netherlands, 2014	Retrospective consecutive cohort design	n = 230	None	NA	NA	The continuation rate after 12 months was 72%; after 24 months, 53%; and after 36 months, 25%, with all women concerned having a new implant placed	1, 2, 3 years	[69]
Sznajder et al., USA, 2016	Retrospective cohort data	n = 160 (12–24 years age)	LNG-IUS and ENG implant	NA	NA	Dis-continuation rate at one year for ENG implant was 22.68%	1 year	[68]
Howard et al., USA, 2017	Retrospective cohort study	Retrospective cohort study based on billing records from a large multispecialty private practice in Las Vegas, Nevada	None	NA	NA	There was a progressive decrease in the 12-month continuation of implant from 2013 (95.7%) to 2015 (57.7%)	1 year	[61]
Aisien et al., Nigeria, 2010	Prospective Longitudinal	n = 32	None	100%	Oligomenorrhea: 18 (56.3%) Menorrhagia: 1(3.1%) and Combination: 13 (40.6%) Headache: 4 (12.5%) and Reduced libido: 3 (9.4%)	Continuation rate at 1 year for ENG implant was 93.8%	12 months	[27]
Weisberg et al., Australia, 2014	Prospective Longitudinal	n = 349	LNG-IUS and ENG implant	NA	NA	47% of implant users discontinued within three years	3 years	[70]
Rominski et al., Ghana, 2018	Pilot longitudinal study	–	Implant, IUD, Injection and Pill	NA	NA	Continuation rate at one year for ENG implant was 95%	1 year	[65]
Arribas et al., Spain, 2009	Prospective	n = 356	None	100%	NA	Continuation rates were 91.0% at 1 year, 74.7% at 2 years and 65.1% at 2 years and 9 months	1, 2, 2 year 9 months	[28]
Boas et al., Brazil, 2016	Prospective cohort study	n = 213 healthy women	None	NA	Increase in weight (63.3–66.1) and BMI (24.7–25.7) and a decrease in TC (172–161.5), TG (75–69.5), and LDL (100.5–98.5) (p > 0.05). Of the metabolic variables, FBG (85–88) and HDL (53–46) had significant differences (p < 0.002)	NA	1, 2, 3 years	[47]
Guazelli et al., Brazil, 2010	Prospective study	n = 47	None	NA	Increase in mean hemoglobin, hematocrit and indirect bilirubin concentrations and of the HDL-C/TC and HDL-C/LDL-C ratio. Decrease in mean TC level as well as LDL-C, very low-density lipoprotein cholesterol, TG, SGOT and SGPT	NA	1 year	[46]
Hidalgo et al., Brazil, 2006	Prospective study	n = 344	LNG implant, Cu-IUD	NA	Implanon, Jadelle and Cu-IUD, 12-month ovarian cyst incidence rates were 26.7%, 14.6% and 1.2%. They regress spontaneously and do not require further treatment	NA	1 year	[52]
Iversen et al., Denmark, 2018	Prospective cohort study	n = 1,879,227	Hormonal contraception	NA	Use of progestogen-only products was not associated with ovarian cancer risk	NA	20 years	[50]
K Gezginc et al., Turkey, 2007	Prospective study	n = 80	None	100%	Amenorrhoea, infrequent bleeding and frequent bleeding were reported by 33(41.25%), 19 (23.75%) and 14 women (17.5%)	25% discontinuation in the study period of 2 years	2 years	[29]
Lidegaard et al., Denmark, 2012	Prospective cohort	n = 1,626,158	OCP, LNG-IUD, Vaginal Ring, Patch and Implant	NA	No significant increase in the risk of thrombotic stroke or myocardial infarction	NA	15 years	[51]
Modesto, Brazil, 2014	Prospective cohort	n = 150	Cu-IUD, ENG-implant	NA	No significant differences on BMD. Increase in body weight (p 0.001) and an increase of 2% in the percentage of body fat, when compared with Cu-IUD users. Increase in lean mass (p = 0.020)	NA	1 year	[44]
Morch et al., Denmark, 2017	Prospective cohort study	n = 1.8 million	OCP, LNG-IUD, Vaginal Ring, Patch and Implant	NA	As compared with never users of contraception, relative risk of breast cancer among implant users was 0.93 (0.48–1.79)	NA	Average of 10.9 years	[49]
Winner et al., USA, 2007	Prospective cohort	n = 7486	DMPA and PPR (Pill, patch, ring)	0.27 per 100 participant-years in LARC as compared to 0.22 in DMPA and 4.55 in PPR	NA	NA	3 years for first 5090 participants and 2 years for those after	[34]
Birgisson et al., USA, 2015	Observational Cohort	n = > 9000	LNG IUS, Cu-IUD and Etonogestrel implant, Patch, Pill and Ring	Combined LARC (LNG-IUS, Copper- IUD and implant) failure rate at 1, 2 and 3 years was 0.3%, 0.6% and 0.9% as compared to 4.8%, 7.8% and 9.4% among short acting contraceptive users	NA	12 and 24 month continuation rate was 83% and 68%	1, 2 years	[39]
Cea Soriano et al., UK, 2014	Prospective Observational study	–	Cu-IUD, LNG-IUS and DMPA injections	NA	NA	13.2% of ENG Implant users discontinued in first year of use	1 year	[56]
Short et al., 4 EU countries, 2012	Prospective Observational study	n = 311	LNG-IUS and ENG implant	NA	NA	Continuation rate at 1 year for ENG implant was 86%	1 year	[67]
Randomised controlled trial								
Apter 2016	RCT	Multicentre RCT n = 766	LNG-IUS and ENG implant	The PIs for LNG-IUS and the ENG implant were 0.9 (95% CI 0.2–2.6) and 0.0 (95% CI 0.0–1.2), respectively	The incidence of adverse events was 84.3% and 79.5% in the LNG-IUS 8 and the ENG implant groups, respectively. In LNG-IUS users: Dysmenorrhea (33.5%), uterine spasms (16.2%), procedural pain (13.6%), headache (11.3%), and acne (9.9%). In ENG implant users, acne (15.5%), headache (12.3%), dysmenorrhea (12.3%), nasopharyngitis (9.2%), and cervical dysplasia (8.9%)	The 12-month discontinuation rates were 19.6% and 26.8% in the LNG-IUS 8 and ENG implant groups, respectively	1 year	[35]
Flores et al., Mexico, 2005	Multicenter trial	n = 417	None	The Pearl Index score was 0	NA	The continuation rate at the end of 3 years was 61.4%	3 years	[25]
Bahamondes et al., Multicentre-seven countries, 2015	Open parallel group RCT	n = 2963	Copper IUD-380A, LNG-implant and ENG implant	0.4 per 100 woman years (CI 0.1–1.4) in ENG implant; 2.8 ( CI 1.3–6.0) in Cu-IUD	ENG implant: Amenorrhea: 38.9%, irregular bleeding: 86.0%, heavy bleeding: 35.38%, prolonged: 56.18%; acne: 45.23%; headache: 59.6% and lower abd pain: 50.35%; dizziness: 44.52% and PID: 1.21% Copper IUD: Amenorrhea: 8.65%, irregular bleeding: 38.93%, heavy bleeding: 49.85%, prolonged: 42.95%; acne: 32.23%; headache: 53.24% and lower abd pain: 61.17% dizziness: 39.96% and PID: 2.68%	Method continuation rates for ENG implant at 2.5 years was 69.8 (95% CI 66.8–72.6)	2.5 years	[37]
Hubacher wt al, USA, 2016	randomized patient preference trial	n = 916	OCP, DMPA, IUD and implant	Implant and IUD (combined) was 0.7 (0.0–4.7) OC: 6.1 (4.3–8.6) DMPA: 4.6 (0.2–12.2)	NA	At one year, probability of continuation of implant was 77.5% (70.2–84.8)	1 year	[36]
Oderich et al., Brazil, 2010	Nonrandomized, open-label, prospective controlled tria	n = 40	Cu IUD and implant	NA	Carbohydrate metabolism was not modified	NA	6, 12 months	[48]
Study design: cross-sectional study								
Nageso et al., Ethiopia, 2018	Cross-sectional study design	n = 711	None	NA	NA	Early Implanon discontinuation rate in this study was 23.4%	1 year	[73]
Gupta et al.,Papua New Guinea, 2017	Cross sectional study	n = 860	None	0.992	Irregular bleeding n = 178, 20.6% but only 7% (n = 13) said the bleeding was bothersome	97% (n = 836) still had the device in situ after 12 months	1 year	[30]
Medhin TG et al., Ethiopia, 2019	Cross sectional study	n = 229	None	NA	NA	Discontinuation: early Implanon discontinuation rate was 38%, 95% CI (32%, 44%).Discontinuation rate was (2.6%) within 6 months, (15.7%) 1 year, (19.7%) in 2 years and (62%) in 3 years	At 6 months, 1, 2 and 3 years	[71]
Smith et al., USA, 2018	Pilot study	n = 29	COCP, Progestin-only contraceptive, non-hormonal or no contraceptive	NA	Progestin-only-contraceptives users have a potential of developing depressive symptoms and have attenuated beta arrestin-1 protein levels in peripheral blood mononuclear leukocytes	NA	Unclear	[76]
Yildizbas et al., Turkey, 2007	A pilot study	n = 41	None	NA	34.1% had amenorrhea; 58.5% had some type of abnormal bleeding. Mood change in 17.1% and acne in 26.8%	NA	6 months	[40]
Study design: retrospective chart review								
Peterson et al., USA, 2019	Retrospective chart review	n = 544	None	NA	NA	Discontinuation rate at one year for ENG implant was 16%	1 year	[64]
Romano et al., USA, 2019	Retrospective chart review	n = 197	Non-users of implant	NA	ENG implant users weight gain + 3.6 (± 7.8) kg vs + 3.1 (± 5.9) kg for controls (P = 0.43). Overall regression analyses showed no group differences among cases and controls	The mean duration of ENG use was 24.5 (± 9.3) months with 21.8% of users (43/197) discontinuing the method early (before 3 years)	20 months	[43]
Mutihir et al., Nigeria, 2010	Retrospective review	n = 669	None	98.51%	NA	95.5% at the end of second year	2 years	[31]
Smith et al., UK, 2002	Retrospective record review and postal survey	n = 190	None	100%	NA	Continuation rates were between 84 and 88% at 6 months and 67% and 78% at 12 months	6, 12 months	[32]
Berlan et al., USA, 2016	Retrospective chart review	n = 750 adolescents	None	NA	NA	77 (10.3%) had the device removed prior to 12 months of use	1 year	[55]
Griffith et al., Australia, 2016	Mixed method study; retrospective chart review	–	Cu-IUD, MPA injection, COCP and ENG-Implant	NA	NA	Etonogestrel continuation rates at 1, 2 and 3 years were 87% (95% CI 81–92%), 72% (95% CI 64–78%) and 51% (95% CI 41–60%) respectively	1, 2, 3 years	[58]
Casey et al., USA, 2010	Retrospective review of medical records	n = 151	None	NA	39 (25.2%) women had abnormal bleeding	Implant removal rate was 25.2% (mean interval, 9.8 months)	90 day reference periods. No fixed follow up	[74]
Harvey et al., Australia, 2009	Retrospective chart	n = 767	None	NA	NA	Continuation at 6 months after insertion was 94%. 74% continued at 1 year, 61% continued at 1.5 years and 50% continued at 2 years.1.2% continued beyond 3 years	Every 6 months up to 3 years	[60]
Sanders et al., USA, 2017	Retrospective chart review	n = 1008	LNG-IUS and Cu-IUD-T380A	NA	NA	Two-year continuation rate 75.9%( 68-mg Etonogestrel implant)	2 years	[66]
Lakha et al., UK, 2006	Case notes Review	n = 324	None	NA	NA	Continuation rates were 89% (CI 84–91) at 6 months, 75% (CI 69–79) at 1 year, 59% (CI 52–63) at 2 years and 47% (CI 40–52) at 2 years and 9 months	Every 6 months upto 3 years	[62]
Agrawal et al., UK, 2005	Case note-based study	n = 106	None	100%	NA	The continuation rate at the end of 1 year was 69.8%, at 2 years was 44.1% and at 3 years was 30.2%	1, 2, 3 years	[26]

### Effectiveness of ESI in comparison with other methods

Of the 15 studies that reported effectiveness of ESI, five of them reported pearl index and the values ranged between 0 and 1.4 unintended pregnancies per 100 woman-years. A multicenter trial from Mexico [25] reported a pearl index of zero with a follow up period of 958.5 woman years. Eight other studies that had no comparator(s) reported effectiveness of 98.5%, 99.2% and the rest 100% respectively [26–33]. Winner et al. compared the effectiveness of long acting reversible contraceptives (LARC: Copper IUD and ESI combined) to that of combined group Oral contraceptive pills, patch, vaginal ring and to Depot Medroxy Progesterone acetate (DMPA) in U.S.A and found that the LARC group had a failure rate of 0.3 per 100 woman years. The group of oral contraceptive pills, patch and vaginal ring had a pooled failure rate of 4.6 per 100 woman years and DMPA had a failure rate of 0.2 per 100 woman years [34]. The Winner et al. study did not report individual method wise effectiveness and instead reported grouped results for LARC and pills, patch and ring. A multicenter randomized control trial in Sweden reported a pearl index of 0.0 (95% CI 0–1.2) in ESI as compared to 0.9 (95% CI 0.2–2.6) in LNG-IUS [35]. Another study, a randomized patient preference trial in U.S.A that recruited 916 women, reported that as compared to grouped short acting reversible contraceptive SARC (pills and DMPA); grouped LARC (Copper IUD and ESI) had a significantly lower probability of unintended pregnancy (6.7 (3.6–12.1) in SARC versus 0.7 (0.0–4.7) in LARC) [36]. A multi-country RCT compared ESI and LNG-implants to Copper-IUD. They found ESI to have a lower failure rate: 0.4 per 100 woman years 95% confidence interval (CI 0.1–1.4) as compared to 2.8 (CI 1.3–6.0) in Copper IUD [37]. A study that analyzed insurance claims among 87,294 women in the USA reported an effectiveness of 98.8% of ESI as compared to 98.9% of LNG-IUS [38]. Secondary analysis of data from the contraceptive CHOICE project, done in U.S.A, reported combined LARC (LNG-IUS, Copper-IUD and implant) failure rate at 1, 2 and 3 years as 0.3%, 0.6% and 0.9% respectively; as compared to 4.8%, 7.8% and 9.4% among users of oral contraceptive pill, patch and vaginal ring. The study did not report method-wise failure rates [39]. Heterogeneity was present among studies that reported effectiveness in terms of study design and comparators and hence meta-analysis could not be conducted.

### Side-effects of ESI as compared to other methods

41.2% (n = 21) of the 51 included studies reported side-effects of ESI. Of the 21 studies, 10 reported menstrual abnormalities; four were on metabolic effects, two on cancers, two on weight gain and body fat, and one each on depression, myocardial infarction/stroke and ovarian cysts.

### Menstrual side-effects

A prospective study in Turkey with 80 participants reported amenorrhoea, infrequent menstrual bleeding and frequent menstrual bleeding among 33(41.3%), 19 (23.8%) and 14 women (17.5%), respectively [29]. Another prospective study from Nigeria with 32 women reported oligomenorrhea in 18 (56.3%); menorrhagia in 1(3.1%) and a combination in 13 (40.6%) women [27]. A retrospective clinic based study with 166 participants reported 40.4% with regular menstrual cycle and 30.3% having regular periods for a few months followed by amenorrhea. Prolonged menstrual bleeding was the most common side-effect reported [33]. A cross-sectional study of 860 women in Papua New Guinea had irregular bleeding as the most commonly reported side effect (20.6%) but only 7% said the bleeding was bothersome [30]. In the 90-day reference period of a pilot study from Turkey with 41 women, three of them (7.3%) had regular menstrual cycles, 14 (34.1%) had amenorrhoea and the remaining 24 (58.5%) had at least one type of abnormality in menstrual bleeding [40]. A multicentre retrospective study in Switzerland among 991 women over a median period of 7.4 months; reported infrequent bleeding (28%), amenorrhea (33%), prolonged bleeding (15%), and menometroorrhagia (16%) [41]. In a retrospective review of clinical records from U.S.A with 151 women, 25.2% contacted a health-care provider for abnormal bleeding [42]. A multi-country RCT showed that women with ESI had amenorrhea (38.9%), irregular bleeding (86.0%), heavy bleeding (35.4%) and prolonged bleeding (56.18%) as compared to women with Copper IUD who had a higher frequency of heavy bleeding(49.9%) and much lower frequency of amenorrhea (8.7%), irregular bleeding ( 38.9%) and prolonged bleeding (43.0%) [37].

Menstrual abnormalities have been recorded using different terminologies (infrequent bleeding, amenorrhea, prolonged bleeding, metromenorrhagia, frequent bleeding, oligomenorrhea, menorrhagia and bothersome bleeding) in different studies. Some of these terminologies do overlap. The terms which meant the same were combined and an average was calculated for the group for ESI. ‘Menorrhagia’, ‘increased bleeding’ and ‘heavy bleeding’ were combined and were found to be prevalent among 32.4% of the women. ‘Oligomenorrhea’, ‘irregular bleeding’ and ‘infrequent bleeding’ were combined and found to be 3.0%. ‘Amenorrhea’ and ‘absent bleeding’ were combined and found to be 15.4%. ‘Metrorrhagia’, ‘prolonged bleeding’, ‘dysfunctional uterine bleeding’ and ‘frequent bleeding’ were combined and found to be 23.3%.

### Other short-term side-effects

Headache among users of ESI was reported in four (19.1%) of the 21 studies and frequency ranged from 5% to 59.6% [27, 35, 37, 41]. Reduction in libido was reported in one study at 9.4% [27]. Abdominal pain was reported among ESI users in a multicentre RCT up to 50.4% [37]. Acne was reported in four studies ranging from 11% to 45% [27, 35, 37, 40]. Other reported short-term side-effects were mastalgia, breast tenderness, emotional lability, mood changes and dizziness.

### Metabolic side-effects

Two studies assessed weight gain among ESI users. A study done in U.S.A in 2019 showed no statistical difference in weight gain among users of ESI and non-users [43]. Another study, a prospective cohort study in Brazil of 150 women compared weight gain, body fat percentage and bone mineral density (BMD) among Copper-IUD and ESI users; findings showed that ESI users did not significantly differ in BMD at 12 months of use as compared to non-users. ESI users experienced gain in body weight of 4.1 kg at 12 months (p value: 0.001) as compared to a decrease in 0.1 kg body weight among Cu-IUD users. ESI users also showed a 2% higher gain in the percentage of body fat as compared to Copper-IUD users. Also, lean mass in ESI users significantly increased at 12 months (p = 0.020) [44]. A sub-study of the Contraceptive CHOICE Project considered 427 women for analysis and showed that there was no difference in weight gain among users of ESI, LNG-IUS or DMPA. Of the 427 users, 30.4% were ESI users who gained on an average 2.1 kg (SD = 6.7), 30.4% were LNG-IUS users who gained 1.0 kg (SD = 5.3), 15.7% were DMPA users who gained 2.2 kg (SD = 4.9) and 23.4% were Copper-IUD users who gained 0.2 kg (SD = 5.1) at the end of one year. However, people with darker skin colour were associated with significant weight gain (1.3 kg, 95% confidence interval = 0.2–2.4) as compared to other racial groups [45]. Parity, age, baseline weight and education of women were some of the covariates analysed by the CHOICE sub-study for continuation rate as an outcomes. No significant difference was found between ESI users and LNG-IUS users for these covariates. Two prospective studies from Brazil showed no significant decrease in total cholesterol, triglycerides and LDL, but a significant increase in mean haemoglobin, haematocrit and indirect bilirubin concentrations and of the HDL-C/TC and HDL-C/LDL-C ratios [46, 47]. A non-randomized open label trial from Brazil with 40 participants showed that ESI users did not have abnormalities in carbohydrate metabolism at 6, 12 months, as compared to baseline [48].

### Long-term side effects

Three of the 51 studies reported long term effects of using ESI. A prospective cohort study of 1.8 million women in Denmark looked at incidence of ovarian and breast cancer among hormonal contraceptive users. The findings showed that users of progestogen-only products (including ESI) were not associated with ovarian cancer risk and that as compared with never users of contraception, relative risk of breast cancer among ESI users was 0.9 (0.5–1.8) [49, 50].The same Danish cohort also looked at incidence of myocardial infarction and stroke among hormonal contraceptive users and found that none of the progestin-only products, including the ESI, significantly increased the risk of thrombotic stroke or myocardial infarction [51]. A prospective study from Brazil with 344 women that looked at occurrence of ovarian cysts among ESI users concluded that the occurrence of ovarian cysts or persistent ovarian follicles in users of ESI was a common finding (26.7%) as compared to users of Copper-IUD (1.2%). The cysts became more frequent with time of use; however they regressed spontaneously within a short period of time and required no further treatment [52].

### Continuation rate of ESI as compared to other methods

**Fig. 2:**
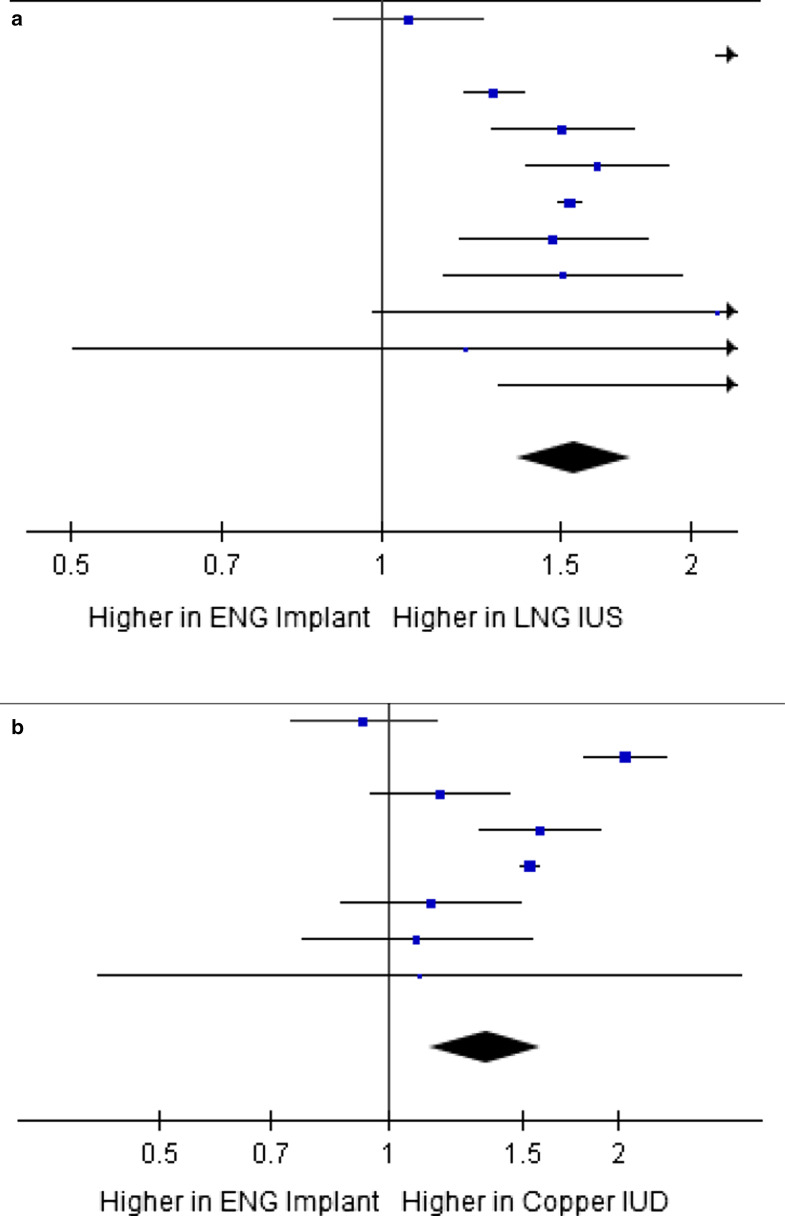
Meta-analyses of one-year continuation rates comparing ESI with LNG-IUS and copper-IUD. **a**, **b** Show the effect size for one year continuation rates for ESI compared to LNG-IUS and Copper-IUD respectively. The square boxes represent effect size of individual studies. The area of the square represents the weight of the study. The horizontal lines represent the limits of 95% confidence interval of the effect sizes. The vertical line is at 1, where there is no difference in continuation rates in the two methods. The diamond shape at the bottom of the plots represents the pooled odds ratio. In both cases it shows that continuation rates are lower in ESI. Source: Generated by authors using RevMan software

19 of the 38 studies that reported continuation rates did not have a comparator. Six of the studies compared ESI with short acting contraceptive methods like oral contraceptive pills, patch or vaginal ring along with at least one long acting method; 12 studies had only long acting contraceptives as comparators like LNG-IUS and Copper-IUD. Our meta-analysis showed that pooled one-year continuation rate was 84.8% for LNG-IUS and 83.0% for Copper-IUD as compared to 77.5% and 76.5% for ESI respectively. Twenty two of the 38 studies reported only continuation rates and not effectiveness/side-effects [42, 53–73]. In the studies, continuation rates varied from 57 to 97% at the end of first year of use, 44–95% at the end of second year and 25–78% by three years of use. A study done in 2010 used the term ‘removal rate’ of ESI. The study reported 25.2% removal rate with a median removal time of 9.8 months [74]. For studies that compared continuation rates of ESI and LNG-IUS and Copper-IUD, meta-analyses were conducted and forest plots were constructed in RevMan software. The meta-analyses presented in Fig. 2 show that odds ratio of one-year continuation rate is 1.6 (1.4, 1.8) for LNG-IUS vs. ESI and 1.3 (1.1, 1.6) for Copper-IUD vs. ESI, thus showing that continuation rates at the end of one-year were higher for LNG-IUS and Copper IUD as compared to ESI. The I^2^ statistic, that measures the variation across studies due to heterogeneity [75], for the two meta-analyses conducted were 90% and 88% respectively, showing that there was high dispersion of effect sizes in the included studies (as the I^2^ was > 75%). Even though the studies were homogenous in their designs and comparators; statistically there was high heterogeneity in the effect sizes. Meta-analysis could not be done for ESI versus DMPA as there was only one study that reported this comparison.

**Fig. 3:**
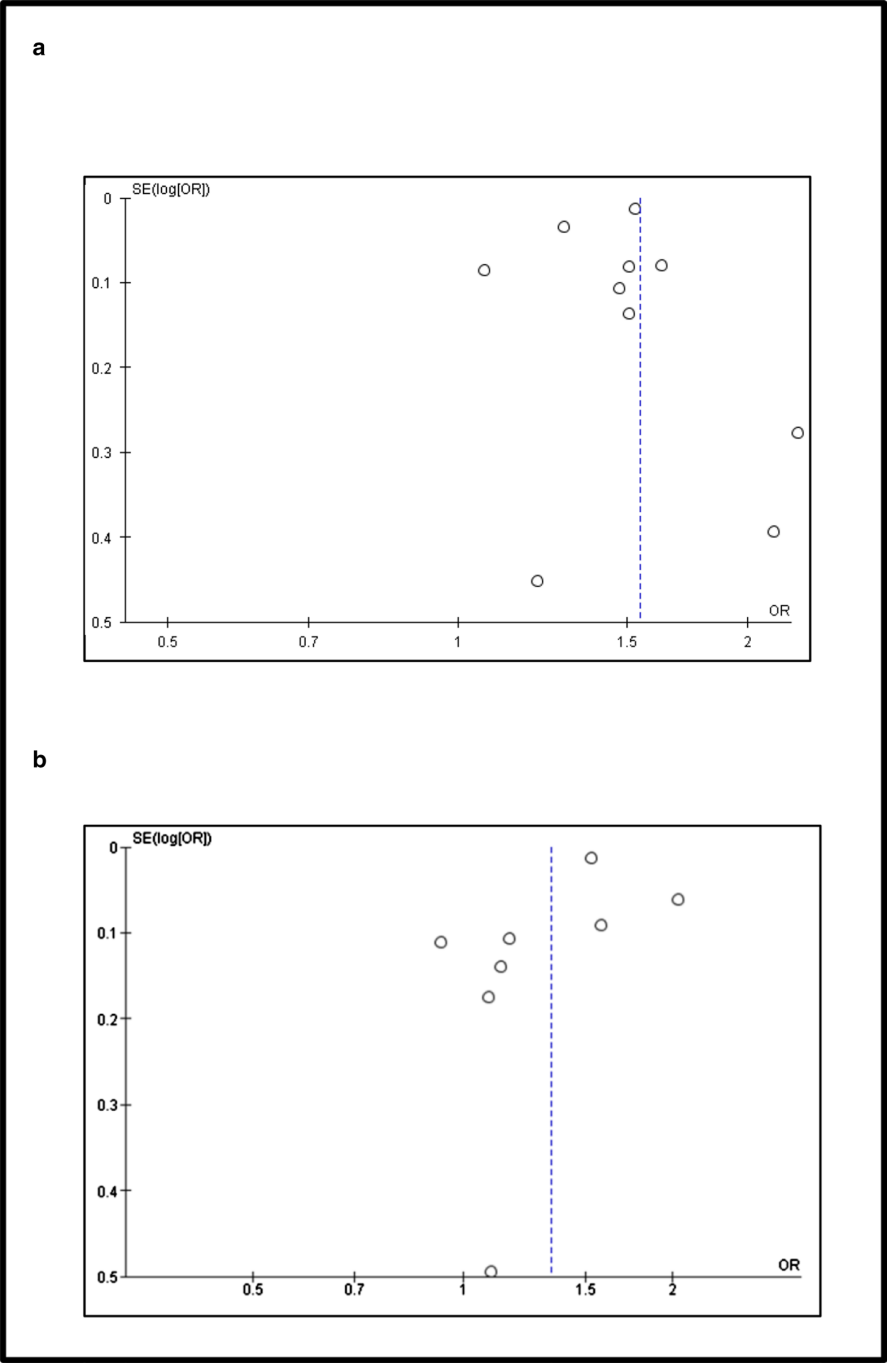
Funnel plots of studies reporting continuation rates comparing ESI with LNG-IUS and Copper-IUD. **a**, **b** Represents the distribution of effect sizes of included studies as a function of standard error comparing ESI with LNG-IUS and Copper-IUD respectively. The asymmetry in **a** and **b** show that there was publication bias. The X-axis plots the odds ratios and the Y-axis plots the standard error. Each hollow circle represents one study. Source: Generated by authors using RevMan software

Reporting bias was assessed for the studies reporting continuation rates and the funnel plots are presented in Fig. 3. The X-axis plots the odds ratios and the Y-axis plots the standard error. Each hollow circle represents one study. The asymmetry in Fig. 3a, b show that there was publication bias.

### Risk of bias and critical appraisal of included studies

The risks of bias of the two included RCTs are presented in Table 2. Both RCTs have high risk of bias due to their open-label nature. One of them had high risk of bias in outcome assessment contributing to detection bias. Both RCTs had incomplete outcome data with attrition bias. The risk of bias in the three non-randomized trials (NRT) is presented in table number 2. Of the three NRTs; two had no clear information on addressing bias due to confounding factors and participant selection. One of the three NRTs has moderate risk of bias and two of them have serious risk of bias, based on the ROBIN-I tool. Based on the CASP tool, 26 (61.9%) of the cohort studies were ‘fair’ in quality; 5 (11.9%) were ‘poor’ and 11 (26.2%) were ‘good’. Of the 42 cohort studies, the common aspects contributing to poor/fair quality were incomplete follow-up, follow-up period being shorter than the three years (ESI use) and no mention of how confounding factors were addressed. Of the four cross sectional studies; all four were of ‘fair’ quality based on AXIS tool. The details of the critical appraisal of the 42 cohort studies and four cross-sectional studies are presented in the Additional file 2.

**Table 2:** Risk of bias in randomized and non-randomized trials

Criteria for Randomized control trials	Dan Apter 2016	David Hubacher 2017
Random sequence generation (selection bias)	Low risk	Low risk
Allocation concealment (selection bias)	Low risk	Low risk
Blinding of participants and personnel (performance bias)	High risk	High risk
Blinding of outcome assessment (detection bias)	High risk	Low risk
Incomplete outcome data (attrition bias)	High risk	High risk
Selective reporting (reporting bias)	Low risk	Low risk
Other bias	NA	NA
Over all	High risk	High risk

Criteria for Non-Randomized control trials	Bahamondes 2015	Flores 2005	Oderich 2012
Bias due to confounding	Moderate risk	NI (No info)	NI (No info)
Bias in selection of participants into the study	Moderate risk	NI (No info)	NI (No info)
Bias in classification of interventions	Low risk	Low risk	Low risk
Bias due to deviations from intended interventions	Low risk	Low risk	Low risk
Bias due to missing data	Moderate risk	NI (No info)	Low risk
Bias in selection of the reported result	Low risk	Low risk	Low risk
Overall	Moderate risk	Serious risk	Serious risk

## Discussion

This systematic review was done in the context of a health technology assessment for etonogestrel subdermal implant (ESI). The protocol for the systematic review was registered in Prospero. We found that ESI was highly effective with pearl index < 1. The short term side-effects included menstrual disturbances (mainly amenorrhea and prolonged bleeding), headache, dizziness, weight gain and acne and were reported by 12 studies of the 51. There were no significant metabolic effects as reported by four studies and no increased risk of long-term adverse-effects like breast cancer, ovarian cancer, myocardial infarction or stroke as reported by two studies. There was an increased incidence of ovarian cysts that was reported by one study but none of the patients required treatment and the cysts regressed on their own after ESI was removed. One study reported that ESI users had a higher potential to develop depression [76]. In comparison to the other LARCs, effectiveness of ESI was the highest. Side-effect profile showed amenorrhea was highest in ESI. Despite the above mentioned advantages, the meta-analyses showed that ESI had lower one-year continuation rates as compared to LNG-IUS and Copper-IUD.

A Cochrane systematic review done in 2007 compared the effectiveness of ESI with other LARC including implants. This review included nine trials, of which eight compared ESI with Norplant (a Levonorgestrel-six-capsule implant) and one compared Jadelle (a Levonorgestrel-1-capsule-implant) with Norplant. The review showed that ESI was highly effective, with no pregnancies reported [77]. A Cochrane systematic review on effect of steroidal contraceptives on BMD included two studies on ESI; one of which fits the inclusion criteria of our review. The review, published in 2014 concluded that fracture risk due to steroidal contraceptives could not be determined from the then existing information [78]. Another Cochrane review that looked at use of progestin-only contraceptives (POC) and weight gain concluded in 2016 that the quality of the available evidence was low and that the 22 included studies showed inconclusive evidence of change in weight or body composition with use of POCs [79].

A systematic review done in 2017 on LARC pooled one-year continuation rates and found that Intrauterine device continuation was 74.0% (95% confidence interval 61.0–87.0%) and implant continuation was 84% (95% confidence interval 77.0–91.0%). This study states as a limitation the heterogeneity among the studies due to which not all continuation outcomes could be combined [80]. This study shows that ESI users have a higher one-year continuation rate as compared to IUD users. This is mainly because the review included women less than 25 years only (it focused on adolescent LARC use). Also, the study includes five studies, till 2016. In comparison, our review includes women of age group 15–49 years and includes studies till the year 2019.

The systematic review shows that ESI is highly effective, yet rate of discontinuation is higher as compared to LNG-IUS and Copper-IID according to our meta-analysis. This is attributed to the side effects of ESI. Of the 38 studies included in our review that report continuation rates; 27 reported reasons for discontinuation of ESI. The most common reasons for discontinuation were menstrual disturbances (6.25–62%) followed by weight gain, mood changes, headache and acne.

There is a huge difference in country representation of studies. As of May 2020, Nexplanon is available for use in 100 countries [81]. Yet, of the 51 included studies; 85% of the studies are from USA, Europe, Australia, Brazil or Mexico (High or upper-middle income countries). Only 7 studies are from low-middle and low income countries, all from Africa. Only one study is from an upper-middle income Asian country: Thailand. Effectiveness of ESI did not vary by country; however, continuation rates at one year were higher in low and low-middle income countries (≥ 85%) as compared to high income countries.

To the best of our knowledge, this is the first systematic review that compares ESI and other long acting reversible contraceptive methods with effectiveness, continuation rates and effects as outcomes. Though our systematic review was able to compare the one-year continuation rates of ESI with LNG-IUS and Copper-IUD by conducting meta-analyses; we were not able to make comparisons in effectiveness and side-effects due to heterogeneity in the studies.

The meta-analyses showed significantly lower one-year continuation rates for ESI as compared to LNG-IUS and Copper-IUD. The reasons for the same have been reported in multiple quantitative studies in literature. A few recent studies indicate reasons for high discontinuation rates among ESI users. A study done in Africa in 2020 reports high likelihood of discontinuation among women living in military camps, having less than three children, no history of previous contraceptive use, having experienced heavy or prolonged bleeding and those who were given poor information about the method [82]. Another study done in Africa in 2017 used mixed methods to determine reasons for discontinuation of implanon. The women who experienced side effects were more likely to discontinue it (Hazard ratio HR 3.6, 95% CI 1.60–8.11). The qualitative part of the study showed that unjustified advice for Implanon removal by non-gynaecologists, due to unrelated users' complaints, and deficient pre-insertion counselling were other causes of early removal.

In our study, of the 21 studies that reported side-effects; 10 reported reasons for discontinuation. Eight studies reported that either prolonged/irregular/increased bleeding were the commonest reasons for discontinuation. Thamkhantho et al. [33] reported that the reason for discontinuation was pain at insertion site (one out of 166). Aisien et al. [27] reported that two out of 32 discontinued one due to menorrhagia and the other due to headache. We have not included the reasons for discontinuation in our review, as that was not our objective; however, we make a call for research to compile evidence on reasons for differences in continuation rates among these long acting contraceptive methods.

Our review included all types of observational studies, including cross-sectional and case-note based studies, which are lower on the evidence pyramid as compared to RCTs. Hence, we calculated sensitivity and precision of our search with and without cross-sectional studies. We found that sensitivity remained unchanged but precision increased when cross-sectional studies were excluded [83].

The quality of included studies is also important in concluding this systematic review. Of the two RCTs, both were open label; attributing to high risk of performance bias. Overall, of the 51 included studies; 78.4% are either fair or poor in quality. The quality of available evidence has to be kept in mind while making policy-level or clinical recommendations about ESI. This will also play an important role in cost-effectiveness analysis involving etonogestrel contraceptive implant.

## Conclusions

We conclude that ESI is safe and effective contraceptive method to use among women of reproductive age (15–49 years). Effectiveness and side-effect rates of ESI could not be pooled in contrast with other LARC due to study heterogeneity. Meta-analyses showed that one-year continuation rates in LNG-IUS were 1.55 (1.36, 1.76) times higher than in ESI users and that in Copper-IUD were 1.34 (1.13, 1.58) times higher than in ESI users. The overall evidence was moderate to low in quality.

## Supplementary Information

**Additional file 1:** Search terms and search strategy. Two tables are included here. The first one lists the search terms and the second lists the search strategy.

**Additional file 2:** Critical appraisal of included studies. Four tables that describe the critical appraisal of included studies.

## Data Availability

All included studies and their critical appraisal details are available in Additional file 2.

## Abbreviations

Cu-IUD: Copper intrauterine device
DMPA: Depot medroxy progesterone acetate
ESI: Etonogestrel subdermal implant
HIV: Human immunodeficiency virus
HR: Hazard ratio
HTA: Health technology assessment
LARC: Long acting reversible contraceptives
LNG-IUS: Levonorgestrel intrauterine system
LH: Luteinizing hormone
RCT: Randomized control trial
SARC: Short acting reversible contraceptive
UK NICE: United Kingdom National Institute for Health and Care excellence
UIP: Unintended pregnancy
WHO: World Health Organization
