# Supplementary material for: A systematic review on clinical effectiveness, side-effect profile and meta-analysis on continuation rate of etonogestrel contraceptive implant
Source: Reprod Health. 2021 Jan 6;18:4. doi: 10.1186/s12978-020-01054-y (PMC7788930; doi:10.1186/s12978-020-01054-y)
Supplement: Supplementary file 1 — Additional file 1: Search terms and search strategy. Two tables are included here. The first one lists the search terms and the second lists the search strategy. [file 12978_2020_1054_MOESM1_ESM.docx]

**Search terms and search strategy**

1. **Search terms used:**

|  | **Key Words (Free text words)** | **MeSH terms** |
| --- | --- | --- |
| **Population** | Women* | Adult  Female  Female, adolescent  Female, adolescents |
| **Intervention** | Implanon  Nexplanon  Implanon-NXT  impalnon-R | agents, reproductive control  agents, contraceptive female  agents, contraceptive  Desogestrel  Hormones, Hormone Substitutes, and Hormone Antagonists  Progestins  Drug implants  etonogestrel |
| **Comparators** | Copper-IUD  LNG-IUS  DMPA | Contraceptive Agents  Contraceptive Agents, Female  Reproductive Control Agents  Levonorgestrel intrauterine device  Intrauterine devices, Copper  Hormones, Hormone Substitutes, and Hormone Antagonists  Progestins  Depot Medroxy progesterone acetate  device, hormone releasing intrauterine  Female sterilization  Tubal ligation |
| **Outcome** | Discontinuation  Continuation  Unintended pregnancy OR Failure rate OR Pearl index | effectiveness, treatment  efficacy, treatment  device safety, medical  Pregnancy  Contraceptive Agents, Female/adverse effects  adverse effects  Side effects  Menstruation/drug effects*  Pregnancy in Adolescence/prevention & control*  Drug implants, adverse effects  adherence, patient |
| **Study designs** | observational study | Randomized Controlled Trial, topics/methods  Non randomized clinical trial, topics/methods  Prospective stud*  Cohort studies  Retrospective stud*  Randomized Controlled Trial  Comparative stud* |

1. **Search strategy in PubMed:**

| Search | Query | Items found |
| --- | --- | --- |
| #50 | Search (((((((((((((((adult[MeSH Terms]) OR adolescent, female[MeSH Terms]) OR adolescents, female[MeSH Terms]) OR women[Text Word]) OR female[MeSH Terms])) AND ((((((((((((agents, contraceptive[MeSH Terms]) OR agents, female contraceptive[MeSH Terms]) OR agents, reproductive control[MeSH Terms]) OR desogestrel[MeSH Terms]) OR etonogestrel[MeSH Terms]) OR progestins[MeSH Terms]) OR (hormones, hormone substitutes, and hormone antagonists[MeSH Terms])) OR drug implants[MeSH Terms]) OR implanon[Text Word]) OR nexplanon[Text Word]) OR implanon-NXT[Text Word]) OR implanon-R[Text Word])) AND (((((((((((((agents, contraceptive[MeSH Terms]) OR agents, female contraceptive[MeSH Terms]) OR agents, reproductive control[MeSH Terms]) OR (hormones, hormone substitutes, and hormone antagonists[MeSH Terms])) OR Levonorgestrel intrauterine device[MeSH Terms]) OR Intrauterine devices, Copper[MeSH Terms]) OR device, hormone releasing intrauterine[MeSH Terms]) OR tubal ligation[MeSH Terms]) OR female sterilization[MeSH Terms]) OR depo medroxyprogesterone acetate[MeSH Terms]) OR copper IUD[Text Word]) OR DMPA[Text Word]) OR LNG-IUS[Text Word])) AND ((((((((((((((((effectiveness, treatment[MeSH Terms]) OR efficacy, treatment[MeSH Terms]) OR device safety, medical[MeSH Terms]) OR pregnancy[MeSH Terms]) OR Contraceptive Agents, Female/adverse effects[MeSH Terms]) OR adverse effects[MeSH Terms]) OR side effects[MeSH Terms]) OR Menstruation/drug effects[MeSH Terms]) OR ("pregnancy in adolescence/prevention and control"[MeSH Terms])) OR "drug implants/adverse effects"[MeSH Terms]) OR adherence, patient[MeSH Terms]) OR discontinuation[Text Word]) OR continuation[Text Word]) OR unintended pregnancy[Text Word]) OR failure rate[Text Word]) OR pearl index[Text Word])) AND ((((((((controlled clinical trials, randomized[MeSH Terms]) OR randomized controlled trials as topic[MeSH Terms]) OR Non randomized clinical trial, topics/methods[MeSH Terms]) OR prospective stud*[MeSH Terms]) OR comparative stud*[MeSH Terms]) OR retrospective stud*[MeSH Terms]) OR cohort stud*[MeSH Terms]) OR observational studies[Text Word])) AND ( "1998/01/01"[PDat] : "2020/03/31"[PDat] ))) NOT endometrio*) NOT diabetes) AND ( "1998/01/01"[PDat] : "2020/03/31"[PDat] )) Filters: Publication date from 1998/01/01 to 2020/03/31 Sort by: [pubsolr12] | 22805 |
| #49 | Search (((((((((((((adult[MeSH Terms]) OR adolescent, female[MeSH Terms]) OR adolescents, female[MeSH Terms]) OR women[Text Word]) OR female[MeSH Terms])) AND ((((((((((((agents, contraceptive[MeSH Terms]) OR agents, female contraceptive[MeSH Terms]) OR agents, reproductive control[MeSH Terms]) OR desogestrel[MeSH Terms]) OR etonogestrel[MeSH Terms]) OR progestins[MeSH Terms]) OR (hormones, hormone substitutes, and hormone antagonists[MeSH Terms])) OR drug implants[MeSH Terms]) OR implanon[Text Word]) OR nexplanon[Text Word]) OR implanon-NXT[Text Word]) OR implanon-R[Text Word])) AND (((((((((((((agents, contraceptive[MeSH Terms]) OR agents, female contraceptive[MeSH Terms]) OR agents, reproductive control[MeSH Terms]) OR (hormones, hormone substitutes, and hormone antagonists[MeSH Terms])) OR Levonorgestrel intrauterine device[MeSH Terms]) OR Intrauterine devices, Copper[MeSH Terms]) OR device, hormone releasing intrauterine[MeSH Terms]) OR tubal ligation[MeSH Terms]) OR female sterilization[MeSH Terms]) OR depo medroxyprogesterone acetate[MeSH Terms]) OR copper IUD[Text Word]) OR DMPA[Text Word]) OR LNG-IUS[Text Word])) AND ((((((((((((((((effectiveness, treatment[MeSH Terms]) OR efficacy, treatment[MeSH Terms]) OR device safety, medical[MeSH Terms]) OR pregnancy[MeSH Terms]) OR Contraceptive Agents, Female/adverse effects[MeSH Terms]) OR adverse effects[MeSH Terms]) OR side effects[MeSH Terms]) OR Menstruation/drug effects[MeSH Terms]) OR ("pregnancy in adolescence/prevention and control"[MeSH Terms])) OR "drug implants/adverse effects"[MeSH Terms]) OR adherence, patient[MeSH Terms]) OR discontinuation[Text Word]) OR continuation[Text Word]) OR unintended pregnancy[Text Word]) OR failure rate[Text Word]) OR pearl index[Text Word])) AND ((((((((controlled clinical trials, randomized[MeSH Terms]) OR randomized controlled trials as topic[MeSH Terms]) OR Non randomized clinical trial, topics/methods[MeSH Terms]) OR prospective stud*[MeSH Terms]) OR comparative stud*[MeSH Terms]) OR retrospective stud*[MeSH Terms]) OR cohort stud*[MeSH Terms]) OR observational studies[Text Word])) AND ( "1998/01/01"[PDat] : "2020/03/31"[PDat] ))) NOT endometrio*) NOT diabetes Filters: Publication date from 1998/01/01 to 2020/03/31 | 22897 |
| #48 | Search (((((((((((((adult[MeSH Terms]) OR adolescent, female[MeSH Terms]) OR adolescents, female[MeSH Terms]) OR women[Text Word]) OR female[MeSH Terms])) AND ((((((((((((agents, contraceptive[MeSH Terms]) OR agents, female contraceptive[MeSH Terms]) OR agents, reproductive control[MeSH Terms]) OR desogestrel[MeSH Terms]) OR etonogestrel[MeSH Terms]) OR progestins[MeSH Terms]) OR (hormones, hormone substitutes, and hormone antagonists[MeSH Terms])) OR drug implants[MeSH Terms]) OR implanon[Text Word]) OR nexplanon[Text Word]) OR implanon-NXT[Text Word]) OR implanon-R[Text Word])) AND (((((((((((((agents, contraceptive[MeSH Terms]) OR agents, female contraceptive[MeSH Terms]) OR agents, reproductive control[MeSH Terms]) OR (hormones, hormone substitutes, and hormone antagonists[MeSH Terms])) OR Levonorgestrel intrauterine device[MeSH Terms]) OR Intrauterine devices, Copper[MeSH Terms]) OR device, hormone releasing intrauterine[MeSH Terms]) OR tubal ligation[MeSH Terms]) OR female sterilization[MeSH Terms]) OR depo medroxyprogesterone acetate[MeSH Terms]) OR copper IUD[Text Word]) OR DMPA[Text Word]) OR LNG-IUS[Text Word])) AND ((((((((((((((((effectiveness, treatment[MeSH Terms]) OR efficacy, treatment[MeSH Terms]) OR device safety, medical[MeSH Terms]) OR pregnancy[MeSH Terms]) OR Contraceptive Agents, Female/adverse effects[MeSH Terms]) OR adverse effects[MeSH Terms]) OR side effects[MeSH Terms]) OR Menstruation/drug effects[MeSH Terms]) OR ("pregnancy in adolescence/prevention and control"[MeSH Terms])) OR "drug implants/adverse effects"[MeSH Terms]) OR adherence, patient[MeSH Terms]) OR discontinuation[Text Word]) OR continuation[Text Word]) OR unintended pregnancy[Text Word]) OR failure rate[Text Word]) OR pearl index[Text Word])) AND ((((((((controlled clinical trials, randomized[MeSH Terms]) OR randomized controlled trials as topic[MeSH Terms]) OR Non randomized clinical trial, topics/methods[MeSH Terms]) OR prospective stud*[MeSH Terms]) OR comparative stud*[MeSH Terms]) OR retrospective stud*[MeSH Terms]) OR cohort stud*[MeSH Terms]) OR observational studies[Text Word])) AND ( "1998/01/01"[PDat] : "2020/03/31"[PDat] ))) NOT endometrio*) NOT diabetes Filters: Publication date from 1998/01/01 to 2020/03/31 Sort by: [pubsolr12] | 22805 |
| #47 | Search (((((((((adult[MeSH Terms]) OR adolescent, female[MeSH Terms]) OR adolescents, female[MeSH Terms]) OR women[Text Word]) OR female[MeSH Terms])) AND ((((((((((((agents, contraceptive[MeSH Terms]) OR agents, female contraceptive[MeSH Terms]) OR agents, reproductive control[MeSH Terms]) OR desogestrel[MeSH Terms]) OR etonogestrel[MeSH Terms]) OR progestins[MeSH Terms]) OR (hormones, hormone substitutes, and hormone antagonists[MeSH Terms])) OR drug implants[MeSH Terms]) OR implanon[Text Word]) OR nexplanon[Text Word]) OR implanon-NXT[Text Word]) OR implanon-R[Text Word])) AND (((((((((((((agents, contraceptive[MeSH Terms]) OR agents, female contraceptive[MeSH Terms]) OR agents, reproductive control[MeSH Terms]) OR (hormones, hormone substitutes, and hormone antagonists[MeSH Terms])) OR Levonorgestrel intrauterine device[MeSH Terms]) OR Intrauterine devices, Copper[MeSH Terms]) OR device, hormone releasing intrauterine[MeSH Terms]) OR tubal ligation[MeSH Terms]) OR female sterilization[MeSH Terms]) OR depo medroxyprogesterone acetate[MeSH Terms]) OR copper IUD[Text Word]) OR DMPA[Text Word]) OR LNG-IUS[Text Word])) AND ((((((((((((((((effectiveness, treatment[MeSH Terms]) OR efficacy, treatment[MeSH Terms]) OR device safety, medical[MeSH Terms]) OR pregnancy[MeSH Terms]) OR Contraceptive Agents, Female/adverse effects[MeSH Terms]) OR adverse effects[MeSH Terms]) OR side effects[MeSH Terms]) OR Menstruation/drug effects[MeSH Terms]) OR ("pregnancy in adolescence/prevention and control"[MeSH Terms])) OR "drug implants/adverse effects"[MeSH Terms]) OR adherence, patient[MeSH Terms]) OR discontinuation[Text Word]) OR continuation[Text Word]) OR unintended pregnancy[Text Word]) OR failure rate[Text Word]) OR pearl index[Text Word])) AND ((((((((controlled clinical trials, randomized[MeSH Terms]) OR randomized controlled trials as topic[MeSH Terms]) OR Non randomized clinical trial, topics/methods[MeSH Terms]) OR prospective stud*[MeSH Terms]) OR comparative stud*[MeSH Terms]) OR retrospective stud*[MeSH Terms]) OR cohort stud*[MeSH Terms]) OR observational studies[Text Word]) Filters: Publication date from 1998/01/01 to 2020/03/31 Sort by: [pubsolr12] | 26491 |
| #46 | Search (((((((((adult[MeSH Terms]) OR adolescent, female[MeSH Terms]) OR adolescents, female[MeSH Terms]) OR women[Text Word]) OR female[MeSH Terms])) AND ((((((((((((agents, contraceptive[MeSH Terms]) OR agents, female contraceptive[MeSH Terms]) OR agents, reproductive control[MeSH Terms]) OR desogestrel[MeSH Terms]) OR etonogestrel[MeSH Terms]) OR progestins[MeSH Terms]) OR (hormones, hormone substitutes, and hormone antagonists[MeSH Terms])) OR drug implants[MeSH Terms]) OR implanon[Text Word]) OR nexplanon[Text Word]) OR implanon-NXT[Text Word]) OR implanon-R[Text Word])) AND (((((((((((((agents, contraceptive[MeSH Terms]) OR agents, female contraceptive[MeSH Terms]) OR agents, reproductive control[MeSH Terms]) OR (hormones, hormone substitutes, and hormone antagonists[MeSH Terms])) OR Levonorgestrel intrauterine device[MeSH Terms]) OR Intrauterine devices, Copper[MeSH Terms]) OR device, hormone releasing intrauterine[MeSH Terms]) OR tubal ligation[MeSH Terms]) OR female sterilization[MeSH Terms]) OR depo medroxyprogesterone acetate[MeSH Terms]) OR copper IUD[Text Word]) OR DMPA[Text Word]) OR LNG-IUS[Text Word])) AND ((((((((((((((((effectiveness, treatment[MeSH Terms]) OR efficacy, treatment[MeSH Terms]) OR device safety, medical[MeSH Terms]) OR pregnancy[MeSH Terms]) OR Contraceptive Agents, Female/adverse effects[MeSH Terms]) OR adverse effects[MeSH Terms]) OR side effects[MeSH Terms]) OR Menstruation/drug effects[MeSH Terms]) OR ("pregnancy in adolescence/prevention and control"[MeSH Terms])) OR "drug implants/adverse effects"[MeSH Terms]) OR adherence, patient[MeSH Terms]) OR discontinuation[Text Word]) OR continuation[Text Word]) OR unintended pregnancy[Text Word]) OR failure rate[Text Word]) OR pearl index[Text Word])) AND ((((((((controlled clinical trials, randomized[MeSH Terms]) OR randomized controlled trials as topic[MeSH Terms]) OR Non randomized clinical trial, topics/methods[MeSH Terms]) OR prospective stud*[MeSH Terms]) OR comparative stud*[MeSH Terms]) OR retrospective stud*[MeSH Terms]) OR cohort stud*[MeSH Terms]) OR observational studies[Text Word]) Sort by: [pubsolr12] | 30713 |
| #45 | Search Sort by: {last_sort_schema} | 0 |
| #44 | Search #42 Filters: Publication date from 1998/01/01 to 2020/03/31 | 22137 |
| #42 | Search ((#41) NOT endometri*) NOT diabetes Filters: Publication date from 1998/01/01 to 2020/03/31 Sort by: [pubsolr12] | 22137 |
| #41 | Search ((((#35) AND #36) AND #37) AND #38) AND #39 Filters: Publication date from 1998/01/01 to 2020/03/31 Sort by: [pubsolr12] | 26480 |
| #40 | Search ((((#35) AND #36) AND #37) AND #38) AND #39 Sort by: [pubsolr12] | 30688 |
| #39 | Search (((((((controlled clinical trials, randomized[MeSH Terms]) OR randomized controlled trials as topic[MeSH Terms]) OR Non randomized clinical trial, topics/methods[MeSH Terms]) OR prospective stud*[MeSH Terms]) OR comparative stud*[MeSH Terms]) OR retrospective stud*[MeSH Terms]) OR cohort stud*[MeSH Terms]) OR observational studies[Text Word] Sort by: [pubsolr12] | 2126331 |
| #38 | Search (((((((((((((((effectiveness, treatment[MeSH Terms]) OR efficacy, treatment[MeSH Terms]) OR device safety, medical[MeSH Terms]) OR pregnancy[MeSH Terms]) OR Contraceptive Agents, Female/adverse effects[MeSH Terms]) OR adverse effects[MeSH Terms]) OR side effects[MeSH Terms]) OR Menstruation/drug effects[MeSH Terms]) OR ("pregnancy in adolescence/prevention and control"[MeSH Terms])) OR "drug implants/adverse effects"[MeSH Terms]) OR adherence, patient[MeSH Terms]) OR discontinuation[Text Word]) OR continuation[Text Word]) OR unintended pregnancy[Text Word]) OR failure rate[Text Word]) OR pearl index[Text Word] Sort by: [pubsolr12] | 2042370 |
| #37 | Search ((((((((((((agents, contraceptive[MeSH Terms]) OR agents, female contraceptive[MeSH Terms]) OR agents, reproductive control[MeSH Terms]) OR (hormones, hormone substitutes, and hormone antagonists[MeSH Terms])) OR Levonorgestrel intrauterine device[MeSH Terms]) OR Intrauterine devices, Copper[MeSH Terms]) OR device, hormone releasing intrauterine[MeSH Terms]) OR tubal ligation[MeSH Terms]) OR female sterilization[MeSH Terms]) OR depo medroxyprogesterone acetate[MeSH Terms]) OR copper IUD[Text Word]) OR DMPA[Text Word]) OR LNG-IUS[Text Word] Sort by: [pubsolr12] | 1327187 |
| #36 | Search (((((((((((agents, contraceptive[MeSH Terms]) OR agents, female contraceptive[MeSH Terms]) OR agents, reproductive control[MeSH Terms]) OR desogestrel[MeSH Terms]) OR etonogestrel[MeSH Terms]) OR progestins[MeSH Terms]) OR (hormones, hormone substitutes, and hormone antagonists[MeSH Terms])) OR drug implants[MeSH Terms]) OR implanon[Text Word]) OR nexplanon[Text Word]) OR implanon-NXT[Text Word]) OR implanon-R[Text Word] Sort by: [pubsolr12] | 1315774 |
| #35 | Search ((((adult[MeSH Terms]) OR adolescent, female[MeSH Terms]) OR adolescents, female[MeSH Terms]) OR women[Text Word]) OR female[MeSH Terms] Sort by: [pubsolr12] | 10771438 |
| Search | Query | Items found |
| #24 | Search #17 Filters: Publication date from 2004/01/01 to 2020/03/31 Sort by: [pubsolr12] | 18773 |
| #17 | Search ((#16) NOT post partum[Title]) NOT post abortion[Title] Filters: Publication date from 2004/01/01 to 2020/03/31 Sort by: [pubsolr12] | 18773 |
| #16 | Search (#11) NOT HIV[Title] Filters: Publication date from 2004/01/01 to 2020/03/31 Sort by: [pubsolr12] | 18800 |
| #13 | Search #11 Filters: Publication date from 2004/01/01 to 2020/03/31 Sort by: [pubsolr12] | 18903 |
| #11 | Search #8 Filters: Publication date from 2004/01/01 to 2020/03/31 Sort by: [pubsolr12] | 18903 |
| #10 | Search #8 Sort by: [pubsolr12] | 26154 |
| #9 | Search (((#1) AND #2) AND #3) AND #5 Sort by: [pubsolr12] | 174846 |
| #8 | Search ((#7) NOT endometr*) NOT diabetes Sort by: [pubsolr12] | 26154 |
| #7 | Search ((((#1) AND #2) AND #3) AND #5) AND #6 Sort by: [pubsolr12] | 31217 |
| #6 | Search ((((((((controlled clinical trials, randomized[MeSH Terms]) OR clinical trials, randomized[MeSH Terms]) OR randomized controlled trial[MeSH Terms]) OR prospective stud*[MeSH Terms]) OR cohort stud*[MeSH Terms]) OR Non randomized clinical trial, topics/methods[MeSH Terms]) OR comparative stud*[MeSH Terms]) OR retrospective stud*[MeSH Terms]) OR observational stud*[Text Word] Sort by: [pubsolr12] | 2176954 |
| #5 | Search (((((((#4) OR adherence, patient[MeSH Terms]) OR side effects[MeSH Terms]) OR discontinuation[Text Word]) OR continuation[Text Word]) OR failure rate[Text Word]) OR pearl index[Text Word]) OR unintended pregnancy[Text Word] Sort by: [pubsolr12] | 2041030 |
| #4 | Search ((((((((effectiveness, treatment[MeSH Terms]) OR efficacy, treatment[MeSH Terms]) OR device safety, medical[MeSH Terms]) OR pregnancy[MeSH Terms]) OR adverse effects[MeSH Terms]) OR "contraceptive agents, female/adverse effects"[MeSH Terms]) OR ("pregnancy in adolescence/prevention and control"[MeSH Terms])) OR drug effects[MeSH Terms]) OR "menstruation/drug effects"[MeSH Terms] Sort by: [pubsolr12] | 1919531 |
| #3 | Search (((((((((((agents, contraceptive[MeSH Terms]) OR agents, female contraceptive[MeSH Terms]) OR agents, reproductive control[MeSH Terms]) OR levonorgestrel[MeSH Terms]) OR copper intrauterine device*[MeSH Terms]) OR tubal ligation[MeSH Terms]) OR female sterilization[MeSH Terms]) OR depo medroxyprogesterone acetate[MeSH Terms]) OR (hormones, hormone substitutes, and hormone antagonists[MeSH Terms])) OR DMPA[Text Word]) OR LNG-IUS[Text Word]) OR Copper IUD[Text Word] Sort by: [pubsolr12] | 1327647 |
| #2 | Search ((((((((((etonogestrel[MeSH Terms]) OR desogestrel[MeSH Terms]) OR agents, contraceptive[MeSH Terms]) OR agents, female contraceptive[MeSH Terms]) OR agents, reproductive control[MeSH Terms]) OR drug implants[MeSH Terms]) OR (hormones, hormone substitutes, and hormone antagonists[MeSH Terms])) OR progestins[MeSH Terms]) OR implanon[Title/Abstract]) OR nexplanon[Title/Abstract]) OR implanon-NXT[Title/Abstract] Sort by: [pubsolr12] | 1315480 |
| #1 | Search (((adult[MeSH Terms]) OR female[MeSH Terms]) OR adolescent, female[MeSH Terms]) OR women[Text Word] Sort by: [pubsolr12] | 10766220 |
