# Supplementary material for: A systematic review on clinical effectiveness, side-effect profile and meta-analysis on continuation rate of etonogestrel contraceptive implant
Source: Reprod Health. 2021 Jan 6;18:4. doi: 10.1186/s12978-020-01054-y (PMC7788930; doi:10.1186/s12978-020-01054-y)
Supplement: Supplementary file 2 — Additional file 2: Critical appraisal of included studies. Four tables that describe the critical appraisal of included studies. [file 12978_2020_1054_MOESM2_ESM.docx]

**Critical appraisal of included studies**

1. **Critical appraisal with CASP tool for cohort studies**

**Table 1: Critical appraisal with CASP tool for cohort studies-I**

| Criteria | Arribas A Mir et al., 2009 | Berenson 2015 | Birgisson 2015 | K Gezginc et al, 2007 | Winner et al, 2007 | Morch et al, 2017 | Modesto, 2014 | Vickery et al, 2013 | Boas et al, 2016 | Lidegaard et al, 2012 | Iversen et al,2018 | Abraham M et al, 2015 |
| --- | --- | --- | --- | --- | --- | --- | --- | --- | --- | --- | --- | --- |
| Did the study address a clearly focused Issue | Yes | Yes | yes | Yes | Yes | Yes | Yes | Yes | Yes | Yes | Yes | Yes |
| Was the cohort recruited in an acceptable way? | Yes | No- only those who had insurance cover for 12 months (data from health insurance claims record) | Can’t say | No | Yes | Yes | Can’t say | Yes | Can’t say | Yes | Yes | Yes |
| Was the exposure accurately measured to minimise bias? | Yes | No | yes | Yes | Yes | Yes | Yes | Yes | Yes | Yes | Yes | Yes |
| Was the outcome accurately measured to minimise bias? | Yes | No | Can’t say | Yes | Yes | Yes | Yes | Yes | Yes | Yes | Yes | Yes |
| Have the authors identified all important confounding factors? | Can’t say | No- Limitations due to choice of the cohort | yes | Can’t say | Can’t say | Yes | Can’t say | Yes | No | Yes | Yes | Can’t say |
| Was the follow-up of the subjects complete enough? | Yes | Yes | yes | Yes | Yes | Yes | Yes | Yes | Can’t say | Yes | Yes | Yes |
| Was the follow-up of the subjects long enough? | Yes | Yes | yes | No (1 year instead of 3) | Yes | Yes | No | No | Yes | Yes | Yes | Yes |
| Do you believe in the results? | Yes | Yes | yes | Yes | Yes | Yes | Yes | Yes | Yes | Yes | Yes | Yes |
| Can the results be applied to the local population? | Can’t say | Can’t say | Can’t say | Yes | Yes | Yes | Can’t say | Yes | Yes | Yes | Yes | Yes |
| Do the results of this study fit with other available evidence? | Yes | Can’t say | Yes | Yes | Yes | Can’t say | Can’t say | Yes | Yes | Can’t say | Can’t say | Yes |
| Are there implications of this study for practice? | Yes | Yes | Yes | No | Yes | Yes | Can’t say | Yes | Yes | Yes | Yes | Yes |
| Conclusion | Good | Poor | Fair | Fair | Good | Good | Poor | Good | Fair | Good | Good | Good |

**Table 2: Critical appraisal with CASP tool for cohort studies-II**

| Criteria | Agostini et al, 2018 | Chiles et al, 2016 | Grunloh et al, 2013 | Howard et al, 2017 | Neil O callahan et al, 2013 | Sznajder et al, 2016 | Teunissen et al, 2013 | Weisberg et al, 2013 | Aisien 2010 | Guazeli et al, 2011 | Hidalgo et al, 2006 | Peipert et al, 2011 | Rominski 2018 | Agrawal 2005 | Berlan 2016 |
| --- | --- | --- | --- | --- | --- | --- | --- | --- | --- | --- | --- | --- | --- | --- | --- |
| Did the study address a clearly focused Issue | Yes | Yes | Yes | Yes | Yes | Yes | Yes | Yes | Yes | Yes | Yes | Yes | Yes | Yes | Yes |
| Was the cohort recruited in an acceptable way? | Yes | Yes | Yes | Yes | Yes | Yes | Yes | Yes | Yes | Can’t say | Yes | Yes | Yes | Yes | Yes |
| Was the exposure accurately measured to minimise bias? | Yes | Yes | Yes | Yes | Yes | Yes | Yes | Yes | Yes | Yes | Yes | Yes | Yes | Yes | Yes |
| Was the outcome accurately measured to minimise bias? | Yes | Yes | Yes | Yes | Yes | Can’t say | Yes | Can’t say | Yes | Yes | Can’t say | Yes | Yes | Can’t say | Yes |
| Have the authors identified all important confounding factors? | No | No | Can’t say | Yes | Yes | No | Can’t say | No | Can’t say | Can’t say | Can’t say | Yes | Can’t say | Can’t say | Can’t say |
| Was the follow-up of the subjects complete enough? | Yes | Yes | Yes | Yes | Yes | Yes | Yes | Yes | Yes | Yes | Yes | Yes | Yes | No | Yes |
| Was the follow-up of the subjects long enough? | No | Yes | Yes | No | No | No | Yes | Yes | No | No | No | No | No | Yes | No |
| Do you believe in the results? | Yes | Yes | Yes | Yes | Yes | Yes | Yes | Yes | Yes | Yes | Yes | Yes | Yes | Yes | Yes |
| Can the results be applied to the local population? | Yes | Can’t say | Yes | Can’t say | Yes | Can’t say | Can’t say | Can’t say | Can’t say | No | Can’t say | Yes | Can’t say | Yes | Yes |
| Do the results of this study fit with other available evidence? | Yes | Yes | Yes | Can’t say | Yes | Yes | Yes | Yes | Yes | Can’t say | Can’t say | Yes | Yes | Yes | Yes |
| Are there implications of this study for practice? | Can’t say | Can’t say | Yes | Can’t say | Can’t say | No | Can’t say | Can’t say | Can’t say | Can’t say | Yes | Yes | Can’t say | Can’t say | Can’t say |
| Conclusion | Fair | Fair | Good | Fair | Good | Fair | Fair | Fair | Fair | Poor | Fair | Good | Fair | Fair | Fair |

**Table 3: Critical appraisal with CASP tool for cohort studies-III**

| Criteria | Bitzer 2004 | cea soriano 2014 | Yildizbas 2007 | Thamkantho 2008 | Smith 2002 | Short 2011 | Sara E casey 2017 | Sanders 2017 | Petersen 2019 | Lakha 2006 | Gupta 2017 | Griffith 2016 | Harvey 2009 | Mutihir 2010 |
| --- | --- | --- | --- | --- | --- | --- | --- | --- | --- | --- | --- | --- | --- | --- |
| Did the study address a clearly focused Issue | Yes | Yes | Yes | Yes | Yes | Yes | Yes | Yes | Yes | Yes | Yes | Yes | Yes | Yes |
| Was the cohort recruited in an acceptable way? | Yes | Yes | Yes | Yes | Yes | Yes | Yes | Yes | Yes | Yes | Yes | Yes | Yes | Yes |
| Was the exposure accurately measured to minimise bias? | Yes | Yes | Yes | Yes | Yes | Can’t say | Yes | Yes | Yes | Yes | Yes | Yes | Yes | Yes |
| Was the outcome accurately measured to minimise bias? | Yes | No | Yes | Yes | No | Yes | Yes | Can’t say | No | Can’t say | No | No | No | No |
| Have the authors identified all important confounding factors? | Can’t say | Can’t say | Yes | Can’t say | No | Can’t say | Yes | Yes | No | No | Can’t say | No | Can’t say | No |
| Was the follow-up of the subjects complete enough? | No | Yes | Yes | No | No | No | No | Can’t say | Can’t say | Can’t say | No | No | No | No |
| Was the follow-up of the subjects long enough? | No | No | No | No | No | No | No | Yes | Yes | Yes | Yes | Yes | Can’t say | No |
| Do you believe in the results? | Yes | Yes | Yes | Yes | Yes | Yes | Yes | Yes | Yes | Yes | Yes | Yes | Yes | Yes |
| Can the results be applied to the local population? | Yes | Yes | Can’t say | Can’t say | Can’t say | Yes | Yes | Yes | Can’t say | Can’t say | Yes | Can’t say | Yes | No |
| Do the results of this study fit with other available evidence? | Yes | Yes | Yes | Yes | Yes | Yes | Yes | Yes | Yes | Yes | Yes | Yes | Yes | No |
| Are there implications of this study for practice? | Yes | Yes | Can’t say | Can’t say | Can’t say | Yes | Yes | Can’t say | Can’t say | Can’t say | Can’t say | Can’t say | Can’t say | No |
| Conclusion | Fair | Fair | Fair | Fair | Poor | Fair | Good | Fair | Fair | Fair | Fair | Fair | Fair | Poor |

1. **Critical appraisal AXIS tool for cross sectional studies**

**Table 4: Critical appraisal AXIS tool for cross sectional studies**

| ***Introduction*** | | Casey 2010 USA | Medhin 2019 | Nageso 2018 | Smith 2018 |
| --- | --- | --- | --- | --- | --- |
| 1 | Were the aims/objectives of the study clear? | Yes | Yes | Yes | Yes |
| ***Methods*** | |  |  |  |  |
| 2 | Was the study design appropriate for the stated aim(s)? | No | No | No | Yes |
| 3 | Was the sample size justified? | No | Yes | Yes | No |
| 4 | Was the target/reference population clearly defined? (Is it clear who the research was about?) | Yes | Yes | Yes | No |
| 5 | Was the sample frame taken from an appropriate population base so that it closely represented the target/reference population under investigation? | No | No | Yes | No |
| 6 | Was the selection process likely to select subjects/participants that were representative of the target/reference population under investigation? | No | No | Yes | No |
| 7 | Were measures undertaken to address and categorise non-responders? | NA | NA | Yes | No |
| 8 | Were the risk factor and outcome variables measured appropriate to the aims of the study? | Yes | No | Yes | Yes |
| 9 | Were the risk factor and outcome variables measured correctly using instruments/measurements that had been trialled, piloted or published previously? | Yes | Yes | No | Yes |
| 10 | Is it clear what was used to determined statistical significance and/or precision estimates? (e.g. p-values, confidence intervals) | Yes | Yes | Yes | Yes |
| 11 | Were the methods (including statistical methods) sufficiently described to enable them to be repeated? | No | Yes | Yes | Yes |
| ***Results*** | |  |  |  |  |
| 12 | Were the basic data adequately described? | Yes | Yes | Yes | Yes |
| 13 | Does the response rate raise concerns about non-response bias? | NA | NA | No | NA |
| 14 | If appropriate, was information about non-responders described? | NA | NA | No | NA |
| 15 | Were the results internally consistent? | Yes | Yes | Yes | Yes |
| 16 | Were the results presented for all the analyses described in the methods? | Yes | Yes | Yes | Yes |
| ***Discussion*** | |  |  |  |  |
| 17 | Were the authors' discussions and conclusions justified by the results? | Yes | Yes | Yes | Yes |
| 18 | Were the limitations of the study discussed? | Yes | Yes | Yes | Yes |
| ***Other*** | |  |  |  |  |
| 19 | Were there any funding sources or conflicts of interest that may affect the authors’ interpretation of the results? | No | No | No | No |
| 20 | Was ethical approval or consent of participants attained? | Yes | Yes | Yes | Yes |
| ***Overall*** | | Fair | Fair | Fair | Fair |
